# Effect of (100) and (001) Hexagonal *WO*_3_ Faceting on Isoprene and Acetone Gas Selectivity

**DOI:** 10.3390/s21051690

**Published:** 2021-03-01

**Authors:** Owen O. Abe, Zanlin Qiu, Joerg R. Jinschek, Pelagia-Irene Gouma

**Affiliations:** 1 Department of Materials Science and Engineering, The Ohio State University, Columbus, OH 43202, USA; abe.24@osu.edu (O.O.A.); qiu.512@osu.edu (Z.Q.); jinschek.1@osu.edu (J.R.J.); 2 Department of Mechanical and Aerospace Engineering, The Ohio State University, Columbus, OH 43202, USA

**Keywords:** metal oxide, gas sensing, hexagonal tungsten trioxide, isoprene, acetone, solvothermal, hydrothermal, crystal facet

## Abstract

The hexagonal $WO_{3}$ polymorph, *h*-$WO_{3}$, has attracted attention due to its interatomic channels, allowing for a greater degree of intercalation compared to other $WO_{3}$ polymorphs. Our research group has previously demonstrated *h*-$WO_{3}$ to be a highly sensitive gas sensing material for a flu biomarker, isoprene. In this work, the gas sensing performance of this polymorph has been further investigated in two distinct configurations of the material produced by different processing routes. The first sample was synthesized using $Na_{2}WO_{4}\cdot 2H_{2}O$ and showed (100) faceting. The second sample was synthesized using $WCl_{6}$ and showed (001) faceting. The gas sensing response of the nanostructured films deposited using the (100) textured *h*-$WO_{3}$ sample 1 had a higher response to acetone at 350 °C. The (001) textured *h*-$WO_{3}$ sample 2 favored isoprene at 350 °C. The selectivity of the latter to isoprene is explained in terms of the dangling bonds present on the (001) facets. The tungsten and oxygen dangling bonds present on the (001) plane favor the adsorption of the isoprene molecule over that of the acetone molecule due to the oxygen containing dipole present in the acetone molecule.

## 1. Introduction

$WO_{3}$ is an important semiconducting binary metal oxide that can be found in at least seven different polymorphic configurations. Of these, at least six belong to uniquely distinct crystal systems. In the bulk, the thermodynamically stable polymorphs from low temperature to high temperature are as follows: monoclinic Pc (ε phase) from 0–230 K; triclinic P$\bar{1}$ (δ phase) from 230–290 K; monoclinic P$2_{1/n}$ (γ phase) from 290–600 K; orthorhombic Pmnb (β phase) from 600–1170 K; and tetragonal P4nmm (α phase) from 1010–1170 K [1]. In addition to these polymorphs, there also exists a kinetically stable hexagonal configuration and a kinetically stable cubic configuration [2]. The hexagonal $WO_{3}$ (*h*-$WO_{3}$) is a metastable polymorph of $WO_{3},$ which was first reported in the late 70 s [3]. Due to its hexagonal structure, the *h*-$WO_{3}$ has been shown to possess a large network of interatomic channels along the c-axis [4].

The original synthesis method for obtaining *h*-$WO_{3}$ utilized an acid precipitation method of sodium tungstate dihydrate [3]. For a time, this was the only successful method in forming this material, and it was thought that the residual sodium in the material acted as a structure directing agent necessary for promoting the growth of the hexagonal structure [5]. This was further supported by the inability of numerous researchers to form *h*-$WO_{3}$ from sodium free $H_{x}WO_{y\cdot }$compounds. Over the years, additional researchers have been able to form *h*-$WO_{3}$ from a variety of precursors including ammonium para-tungstate, tungsten hexachloride, and tungsten foils [4,6,7,8]. Furthermore, *h*-$WO_{3}$ has been reported in a variety of different morphologies ranging from platelets and rods to flowers [9]. The functionality of these different morphologies has been studied for a variety of different fields including metal oxide ($MO_{x}$) gas sensors with varying results. Often, researchers attribute their findings to the new improved morphology developed. However, the morphology changes from reference-to-reference, and the gaseous analyte detected also differs; thus, it is difficult to understand the underlying operating mechanism controlling the sensing response with respect to a particular gaseous analyte.

As metal oxide gas sensing is a surface driven phenomenon, typically involving the adsorption of a gaseous analyte onto the surface of the $MO_{x}$, the properties of the exposed surface strongly impact the overall gas sensing performance [10]. Jian et al. demonstrated the significance of sample faceting by studying the adsorption of various organic dyes onto *h*-$WO_{3}$ nanorods synthesized to exhibit (001) and ($1\bar{1}0)$ faceting [9]. In their work, they demonstrated that dye adsorption of the $\left(1\bar{1}0\right)$ material was nearly three times as high as the (001) material [9]. Jia et al. synthesized *h*-$WO_{3}$ nanorods with (002) faceting and (100) faceting for $MO_{x}$ gas sensing towards acetone and ethanol [11]. In their work, they demonstrated that at 280 °C, the response towards ethanol was higher for the (100) faceted nanorods, while the (002) faceted nanorods responded better towards acetone [11]. Wu et al. studied the *h*-$WO_{3}$ (001) and (110) facets using density functional theory (DFT) and found that the (001) facet is insensitive to methane, while the (110) facet allows for a large amount of electron transfer upon exposure due to coordination of the W and O bonds on the surface [12]. Isoprene being a biomarker for flu makes understanding its gas sensing properties even more appealing [13,14,15,16].

To investigate this further, two configurations of *h*-$WO_{3}$ were synthesized, one via a solvothermal process and another using a hydrothermal process. Both synthesis routes produced crystalline nanopowders exhibiting a platelet morphology with one exhibiting primarily with (001) facet texturing while the other exhibiting primarily (100) facet texturing. The gas sensing response of the materials towards acetone and isoprene was studied in tube furnace style gas flow bench. A possible mechanism for the difference in their respective gas sensing responses is provided herein.

## 2. Materials and Methods

An acid precipitation method was used to synthesize the randomly oriented *h*-$WO_{3}$ used in this study [17]. The starting materials consisted of $Na_{2}WO_{4}\cdot 2H_{2}O$ (Sigma Aldrich) and hydrochloric acid, HCL (Alfa Aesar 36.5–38%). The process was as follows: 1.17 g of $Na_{2}WO_{4}\cdot 2H_{2}O$ was dissolved in 17 mL of deionized water, sonicated for 20 min, and then placed in an ice bath and allowed to cool for 60 min. Concurrently, 8.4 mL of HCl (18% in excess of equimolar reaction) was also allowed to cool in an ice bath for 60 min. After several hours, the cooled HCl acid was added to the cooled aqueous $Na_{2}WO_{4}\cdot 2H_{2}O$ mixture in one dose. During this time, the mixture gelated almost instantly and turned white. This solution gradually turned from white to lemon cake yellow, and the mixture was allowed to age for 24 h in an ice bath.

At this point, deionized water was added to the mixture, and it was gently stirred with a glass rod. Afterwards the mixture was centrifuged, and the precipitate was removed and washed several times with deionized water to remove excessive sodium and chloride ions. Afterwards the washed tungstic acid precipitate was allowed to dry overnight, the precipitate was added to a Teflon lined hydrothermal pressure vessel (Parr Instruments 4700 Series 45 mL). The pressure vessel was filled to 90% with deionized water and placed in an oven for twelve hours at 120 °C. During this time, hydrothermal dehydration of the tungstic acid occurred at subcritical autogenous pressures to form $WO_{3}\cdot \frac{1}{3}H_{2}O$ via $WO_{3}\cdot$intraparticle dehydration. The as-received material was centrifuged and washed with deionized water several times and allowed to dry at room temperature in a fume hood. The remaining material was heat treated at 350 °C for two hours to obtain the final *h*-$WO_{3}$ product. 

This acid precipitation route and hydrothermal dehydration can be described by the following series of reactions:

a *Na*_2_*WO*_4 ∙ _ 2*H*_2_*O* dissociation in an alkaline aqueous
  $$Na_{2}{\mathrm{WO}}_{4}\cdot 2H_{2}O\overset{\begin{array}{c} \mathrm{Aqueous}\\ \mathrm{Dissociation} \end{array}}{\to }2Na^{+}+{\left[WO_{4}\right]}^{2-}+2H_{2}O$$
b Precipitation of *H*_2_*WO*_4_ in hydrochloric acid
  $$2Na^{+}+{\left[WO_{4}\right]}^{2-}+H_{2}O+2HCl\overset{\begin{array}{c} \mathrm{Aqueous}\mathrm{Acid}\\ \mathrm{Precipitation} \end{array}}{\to }H_{2}WO_{4}\cdot H_{2}O+2Na^{+}+2Cl^{-}$$
c Self-ionization of water to produce *OH*^−^ + *H*^+^ ions
  $$H_{2}O\overset{\begin{array}{c} \mathrm{Self}\\ \mathrm{Ionization} \end{array}}{\to }OH^{-}+H^{+}$$
d Hydrothermal dehydration of crystalline water by subcritical water
  $$\begin{array}{c} 3\left[H_{2}WO_{4}\cdot H_{2}O\right]+OH^{-}+H^{+}\\ \overset{\begin{array}{c} \mathrm{Hydrothermal}\\ \mathrm{Dehydration} \end{array}}{\to }\\ 3\left[WO_{3}\cdot \frac{1}{3}H_{2}O\right]+6H_{2}O \end{array}$$
e Thermal dehydration of product at 350 °C to form anhydrous ***h*-**WO_3_
  $$WO_{3}\cdot \frac{1}{3}H_{2}O\overset{\begin{array}{c} \mathrm{Thermal}\\ \mathrm{Dehyderation} \end{array}}{\to }h-WO_{3}$$

A chloride based solvolysis method was performed to synthesize the (001) *h*-$WO_{3}$ [18]. Starting materials consisted of $WCl_{6}$ (Sigma-Aldrich; St. Louis, MO, USA) and ethanol anhydrous (Sigma-Aldrich). The solvolysis method was performed in an argon filled glove box to prevent hydration of the $WCl_{6}$ precursor. The process was as follows: 100 mL of ethanol anhydrous was heated to 70 °C under constant magnetic stirring; 3.95 g of $WCl_{6}$ was added to this solution in one dose under magnetic stirring to create a 0.1 M solution of tungsten alkoxychloride. Upon addition of the $WCl_{6}$, the solution immediately turns yellow as HCl vapor is emitted from the container. Eventually the solution turns red, clear, and blue, and ultimately arrives at a deep green color after one week of aging. During this solvolysis process and aging, substitution of the chlorine ligand for the ethoxy ligand results in a change in coordination of the tungsten alkoxychloride, which is reflected in its color.

After the solution has aged for one week, 10 mL of the green tungsten alkoxychloride was removed from the argon filled glovebox and added to 10 mL of distilled water. The mixture immediately turned blue, indicating the onset of hydrolysis of the tungsten alkoxychloride into the dark blue amorphous $WO_{3}$ monohydrate. The colloidal mixture was allowed to age under magnetic stirring for three hours to ensure hydration and then transferred to a Teflon lined hydrothermal pressure vessel (Parr Instruments 4700 Series 45 mL) and heated for twelve hours at 200 °C from room temperature at a ramp rate of 3 °C per min. During this ramping time, hydration of the tungsten alkoxychloride was accelerated via reaction with the existing water and hydrothermal decomposition of the ethoxy groups, further increasing the concentration of water in the system. As the temperature increases, self-ionization of the water occurs, which in turn enables the hydrothermal dehydration of the $WO_{3}$ hydrate via intraparticle dehydration to form $WO_{3}\cdot \frac{1}{3}H_{2}O$. 

The as-received material was light blue, and it was centrifuged and washed with deionized water and ethanol several times, during which the organics washed away and revealed an off-white powder. This powder was allowed to dry at room temperature in a fume hood. The material was then heat treated at 350 °C for 2 h to obtain the final *h*-$WO_{3}$ product. 

This solvolysis route and solvothermal synthesis route can be described be the following series of reactions:

a) Solvolysis of *WCl*_6_ in anhydrous ethanol
  $$WCl_{6}+nC_{2}H_{5}OH\to W\left(OC_{2}H_{5}\right)nCl_{n-6}+nHCl$$
b) Hydration of tungsten alkoxychloride to form *WO*_3_∙*H*_2_*O*
  $$W{\left(OC_{2}H_{5}\right)}_{n}Cl_{n-6}+nHCl+H_{2}O\overset{\mathrm{Hydrolysis}}{\to }WO_{3}\cdot H_{2}O+nHCl+\left(n-6\right)C_{2}H_{5}OH$$
c) Hydrothermal decomposition of ethanol to form excess water and ethylene
  $$C_{2}H_{5}OH\overset{\begin{array}{c} \mathrm{Hydrothermal}\\ \mathrm{Decomposition} \end{array}}{\to }C_{2}H_{4}+H_{2}O$$
d) Self-Ionization of water to produce *OH*^−^ + *H*^+^ ions
  $$H_{2}O\overset{\begin{array}{c} \mathrm{Self}\\ \mathrm{Ionization} \end{array}}{\to }OH^{-}+H^{+}$$
e) Hydrothermal dehydration of crystalline water by subcritical water
  $$3\left[H_{2}WO_{4}\right]+OH^{-}+H^{+}\overset{\begin{array}{c} \mathrm{Hydrothermal}\\ \mathrm{Dehydration} \end{array}}{\to }3\left[WO_{3}\cdot \frac{1}{3}H_{2}O\right]+3H_{2}O$$

Gas sensors were prepared by mixing 0.01 g of the heat-treated *h*-$WO_{3}$ powders with 1 mL of 2-heptanol and drop coating 1 μL of the suspension onto interdigitated electrodes to form the sensing film and allowed to dry overnight. These electrodes consisted of an alumina substrate with interdigitated platinum “fingers” and gold wire leads [19]. Electrical connections to the digital multimeter were established via the gold leads, and the gas sensing response of the sensors were studied using a custom gas flow bench consisting of a tube furnace (Lindberg/Blue), MKS 1179a mass flow controllers, and an MKS 247D power supply. Gas sensing experiments were conducted in 500 sccm OSHA grade D breathing air with a relative humidity of less than 0.1% [19]. The crystal structure of the materials was determined using X-ray powder diffractometry (XRD Bruker D8). The sample morphology was assessed through the use of transmission electron microscopy (TEM FEI Tecnai G2 30 TWIN). Analysis of micrographs were conducted using the ImageJ software.

## 3. Results

### 3.1. Crystal Structure

**Figure 1:**
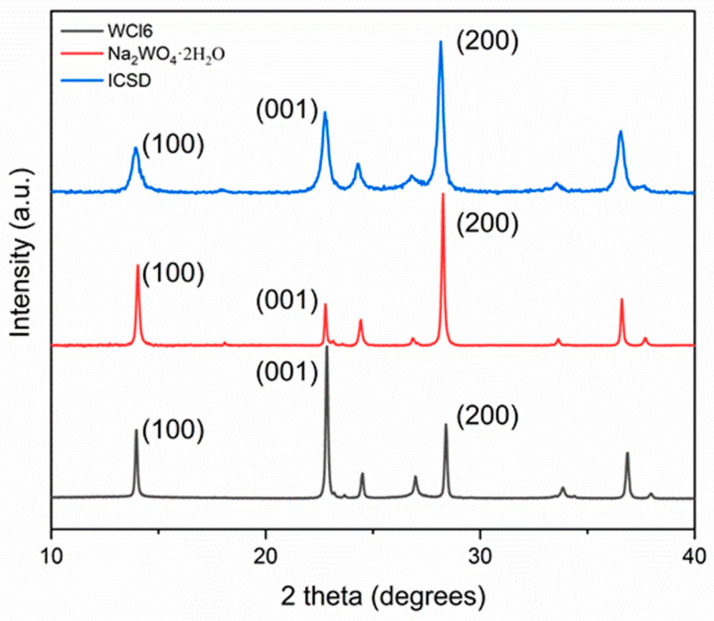
XRD spectra of the standard reference pattern (ICSD 32001 from Ref. [3]) for the hexagonal polymorph of tungsten trioxide (*h*-$WO_{3}$) in comparison to the *h*-WO3 samples synthesized in this work using the $Na_{2}{\mathrm{WO}}_{4}\cdot 2H_{2}O$ and $WCl_{6}$ precursors.

Figure 1 shows the X-ray diffraction (XRD) spectra of the *h*-$WO_{3}$ samples synthesized in this work using the $Na_{2}{\mathrm{WO}}_{4}\cdot 2H_{2}O$ and $WCl_{6}$ precursors. Both patterns are well indexed to the typical diffraction peak profile of the *h*-$WO_{3}$ standard reference pattern (ICSD 32001).

For the $Na_{2}{\mathrm{WO}}_{4}\cdot 2H_{2}O$ sample, the relative intensity of the (100) peak compared with the (001) peak is stronger than what is reported in the standard reference pattern. However, for the $WCl_{6}$ *h*-$WO_{3}$ sample, the relative intensity (001) peak compared with the (100) peak is significantly stronger than what is reported in both the standard reference pattern and the $Na_{2}{\mathrm{WO}}_{4}\cdot 2H_{2}O$ *h*-$WO_{3}$ pattern. This observation is the indicative of crystal facet exposure.

The relative texture coefficient of a specific crystal facet, $TC_{\left(hkl\right)}$, was used to evaluate the degree of crystal facet exposure in the $Na_{2}{\mathrm{WO}}_{4}\cdot 2H_{2}O$ and $WCl_{6}$ synthesized *h*-$WO_{3}$ samples. The texture coefficients of the (001) and (100) facets are given by the following equations [11]:

For the $Na_{2}{\mathrm{WO}}_{4}\cdot 2H_{2}O$ sample, the relative intensity of the (100) peak compared with the (001) peak is stronger than what is reported in the standard reference pattern. However, for the $WCl_{6}$ *h*-$WO_{3}$ sample, the relative intensity (001) peak compared with the (100) peak is significantly stronger than what is reported in both the standard reference pattern and the $Na_{2}{\mathrm{WO}}_{4}\cdot 2H_{2}O$ *h*-$WO_{3}$ pattern. This observation is the indicative of crystal facet exposure.

The relative texture coefficient of a specific crystal facet, $TC_{\left(hkl\right)}$, was used to evaluate the degree of crystal facet exposure in the $Na_{2}{\mathrm{WO}}_{4}\cdot 2H_{2}O$ and $WCl_{6}$ synthesized *h*-$WO_{3}$ samples. The texture coefficients of the (001) and (100) facets are given by the following equations [11]:

$${\mathrm{TC}}_{001}=\frac{I_{001}/I_{001}^{0}}{I_{001}/I_{001}^{0}+I_{100}/I_{100}^{0}}\;{\mathrm{TC}}_{100}=\frac{I_{100}/I_{100}^{0}}{I_{001}/I_{001}^{0}+I_{100}/I_{100}^{0}}$$

where ${\mathrm{TC}}_{001}$ and ${\mathrm{TC}}_{100}$ are the relative texture coefficients of the diffraction peaks of (001) over (100) and (100) over (001), respectively. $I_{001}$ and $I_{100}$ are the measured diffraction peak intensities from the XRD patterns in Figure 1 of the $Na_{2}{\mathrm{WO}}_{4}\cdot 2H_{2}O$ and $WCl_{6}$ synthesized *h*-$WO_{3}$ samples. $I_{001}^{0}$ and $I_{100}^{0}$ are the corresponding values of the standard reference pattern from a randomly oriented sample of *h*-$WO_{3}$. The texture coefficients for the samples can be found in Table 1. From this, it can be seen that the ${\mathrm{TC}}_{001}$ for the $WCl_{6}$ *h*-$WO_{3}$ sample is 0.75, indicating that this sample has mainly (001) facets exposed. The ${\mathrm{TC}}_{100}$ for the $Na_{2}{\mathrm{WO}}_{4}\cdot 2H_{2}O$ sample is 0.66, indicating that this sample has mainly exposed with (100) facets (Note: The texture coefficient of a randomly oriented sample is 0.5, indicating its randomness).

### 3.2. Crystal Morpholgy

**Figure 2:**
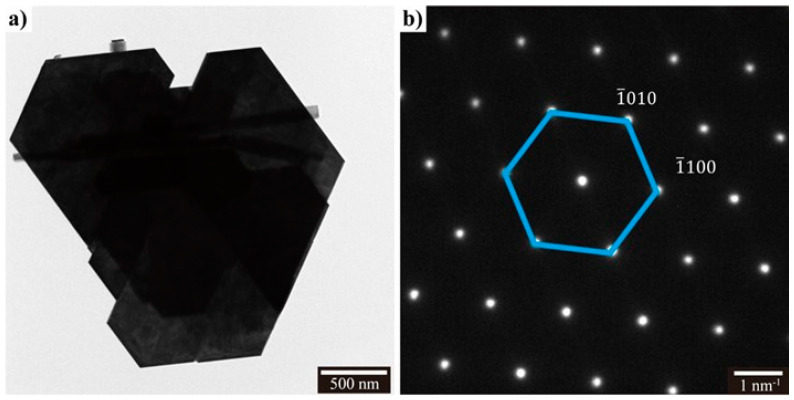
TEM micrograph of the $WCl_{6}$ *h*-$WO_{3}$ (**a**) and SAED pattern (**b**).

Transmission electron microscopy (TEM) micrographs of a crystallite from the $WCl_{6}$ *h*-$WO_{3}$ sample can be seen in Figure 2a and consist of large flat faceted platelets with length scales on the order of hundreds of nanometers. The hexagonal features of the sample can clearly be seen by the ≈120° crystal edges. Selected area electron diffraction (SAED) patterns of the $WCl_{6}$ *h*-$WO_{3}$ crystallite can be seen in Figure 2b. These patterns show the material to be highly defect free and to correspond with the [001] zone axis of the hexagonal polymorph of $WO_{3}$. These results further indicate that the large facet observed in Figure 2a corresponds with the (001) surface. 

Figure S1a shows the morphology of the $Na_{2}{\mathrm{WO}}_{4}\cdot 2H_{2}O$ sample. The morphology of it is a combination of nanoplates and nanorods (See Supplement). In the Figure S1b, nanoplates with some “feelers” are observed, which suggests that the nanoplates are formed by aggregation of the nanorods. Although some nanorods are present in Figure 2a, crystallites with “feeler” features are not observed. Based on that, it is reasonable to deduce that the 001 facets plates are not the aggregation mechanism mentioned above. 

### 3.3. Gas Sensing

**Figure 3:**
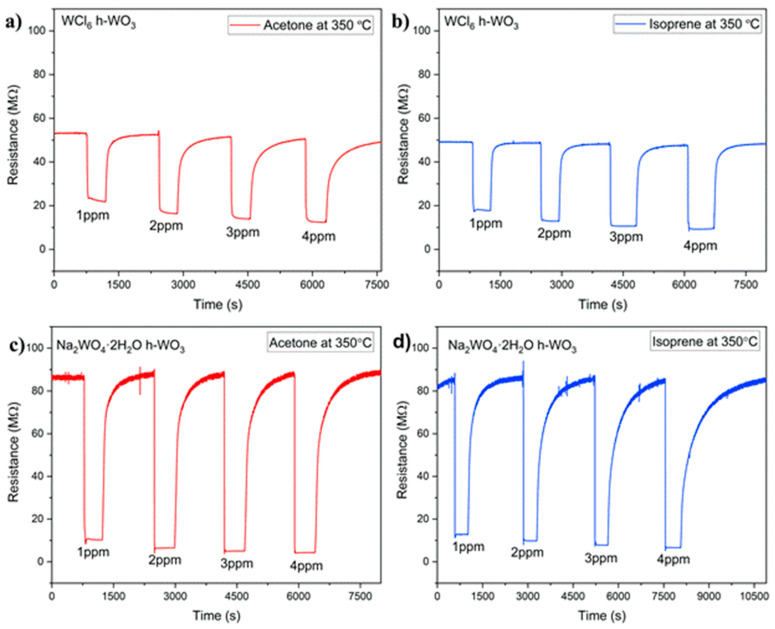
Sensing response of the $WCl_{6}$ *h*-$WO_{3}$ sample towards 1, 2, 3, and 4 ppm acetone (**a**) and isoprene (**b**) at 350 °C. Sensing response of the $Na_{2}{\mathrm{WO}}_{4}\cdot 2H_{2}O$ *h*-$WO_{3}$ sample towards 1, 2, 3, and 4 ppm acetone (**c**) and isoprene (**d**) at 350 °C.

**Figure 4:**
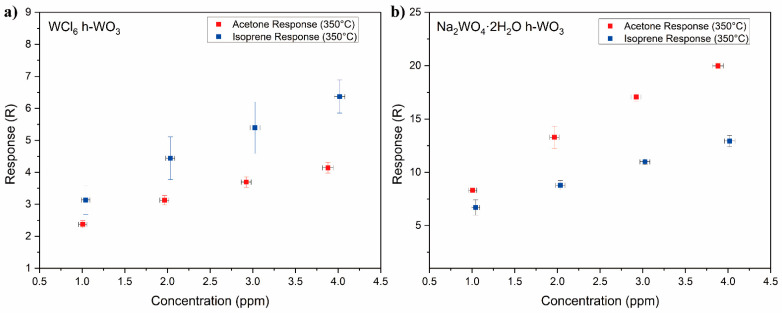
Comparison of the gas sensing response of the $WCl_{6}$ *h*-$WO_{3}$ sample (**a**) and the $Na_{2}WO_{4}\cdot 2H_{2}O$ sample towards acetone and isoprene at 350 °C for 1, 2, 3 and 4 ppm gas concentrations (**b**) along with their standard deviations.

Figure 3a,b show the sensing response at 350 °C for the $WCl_{6}$* h*-$WO_{3}$ sample towards 1, 2, 3 and 4 ppm of acetone (a) and isoprene (b) gas, respectively Figure 3c,d show the sensing response at 350 °C for the $Na_{2}{\mathrm{WO}}_{4}\cdot 2H_{2}O$ *h*-$WO_{3}$ sample towards 1, 2, 3, and 4 ppm acetone (c) and isoprene (d) gas, respectively. The response (R) of the sensors have been calculated using the following equation:

Response (*R*) = *R_O_/R_G_*

where $R_{0}$ is taken to be the baseline resistance of the sensor and was measured at 350 °C in 500 sccm OSHA grade D breathing air. $R_{g}$ is taken to be the resistance of the sensor upon exposure to the test gas. In this work, the test gasses are isoprene and acetone. Response vs. concentration plots for the $WCl_{6}$ *h*-$WO_{3}$ and $Na_{2}{\mathrm{WO}}_{4}\cdot 2H_{2}O$ *h*-$WO_{3}$ samples towards acetone and isoprene at 350 °C can be seen in Figure 4a,b, respectively. Figure 4 shows that the response of the $WCl_{6}$ *h*-$WO_{3}$ consistently exhibited a higher response towards isoprene at every test concentration. This differs from the $Na_{2}{\mathrm{WO}}_{4}\cdot 2H_{2}O$ *h*-$WO_{3}$, which exhibited a higher response towards acetone at every test concentration.

**Figure 5:**
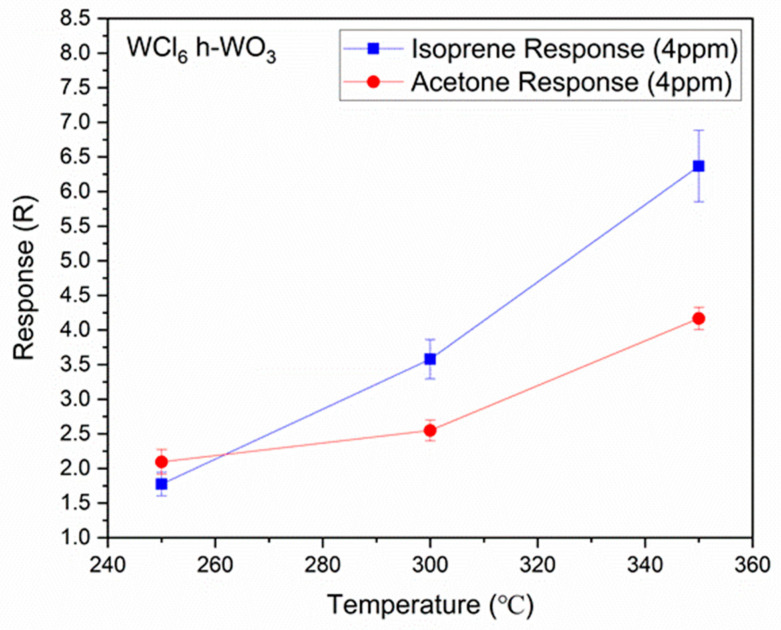
Comparison of the gas sensing response for the $WCl_{6}$ *h*-$WO_{3}$ nanstructured films from Figure 3 at 250, 300, and 350 °C towards 4 ppm of acetone and isoprene along with their standard deviations.

To evaluate what the optimal operating temperature is for the sensing material, the response to acetone and isoprene was also evaluated at 250 and 300 °C (in addition to 350 °C) using similar acetone and isoprene concentrations. A plot of the gas sensor response, R, as a function of temperature for the $WCl_{6}$ *h*-$WO_{3}$ can be seen in Figure 5. 

## 4. Discussion

### Crystal Morpholgy

The primary difference between the two synthesis routes for *h*-$WO_{3}$ lies in the chemistry of the precursors used, the details of which are unique to the synthesis routes taken, but the final step is always to obtain the $WO_{3}\cdot \frac{1}{3}H_{2}O$ percussor. The orthorhombic $WO_{3}\cdot \frac{1}{3}H_{2}O$ is an intermediate phase of *h*-$WO_{3}$. When subjected to mild thermal heat treatments, this material transforms into an anhydrous *h*-$WO_{3}$. When this was initially discovered by Gerand et al., it was not exactly clear as to why the 13 hydrate is the only hydrate of WO_3_ that forms *h*-$WO_{3}$. Work by the author and work reported by other researchers have reported the inability for the monohydrate (1·$H_{2}O$) or dihydrate (2·$H_{2}O$) to form *h*-$WO_{3}$ when subjected to mild thermal heat treatments [3,20]. Today, it is thought to be uniquely related to the crystal structure of the 13 hydrate compared to that of the higher order hydrates [21,22]. The crystal structure of the monohydrate and dihydrate consists of corner sharing octahedra with layers of interatomic water alternating with layers of $WO_{6}$ octahedrons [23]. The dihydrate compares to the monohydrate by the existence of two interatomic water molecules. 

The 13 hydrate is also orthorhombic and contains interatomic water molecules, but it is coordinated with $WO_{6}$ octahedrons and $WO_{6}\cdot H_{2}O$ octahedrons, which average out to an overall hydration composition of 13 [21]. This results in a change in the coordination of the octahedrons, causing a lateral shift of the octahedrons in the a/b plane forming the primary framework for the *h*-WO_3_ along the c-axis [22]. When the 13 hydrate is subjected to a mild heat treatment, the orthorhombic $WO_{3}\cdot \frac{1}{3}H_{2}O$ transforms into the anhydrous *h*-$WO_{3}$[21,22].

The primary difference between the two anhydrous samples is the large relative intensity of the 001 peak in the $WCl_{6}$ *h*-$WO_{3}$ sample compared to that of the reference sample or the $Na_{2}{\mathrm{WO}}_{4}\cdot 2H_{2}O$ *h*-$WO_{3}$ prepared in this work. As expected, the TEM micrographs in Figure 2. showed that the large flat surface in the $WCl_{6}$ *h*-$WO_{3}$ crystallite is the (001) surface, and the viewing direction is the [001] direction of the *h*-$WO_{3}$ structure. This (001) facet texturing is the reason for the anomalously high relative intensity of the 001 peak in the XRD pattern reported in Figure 1. The texturing coefficient of the $WCl_{6}$ *h*-$WO_{3}$ (001) facet was determined to be 0.75 further, indicating the presence of a highly oriented (001) faceted *h*-$WO_{3}$. The texturing coefficient of the $Na_{2}{\mathrm{WO}}_{4}\cdot 2H_{2}O$ *h*-$WO_{3}$ sample was 0.66 for the (100) facet, further indicating a preferred texting in this sample which differs from the $WCl_{6}$ *h*-$WO_{3}$. 

Examining Figure 4, it can be readily observed that the response of the $Na_{2}{\mathrm{WO}}_{4}\cdot 2H_{2}O$ *h*-$WO_{3}$ was consistently higher for acetone than isoprene at every concentration tested. Compared with that of the $Na_{2}{\mathrm{WO}}_{4}\cdot 2H_{2}O$ *h*-$WO_{3}$, the gas sensing response of the $WCl_{6}$ *h*-$WO_{3}$ was lower overall at every concentration of each gas. Additionally, the response of the (001) *h*-$WO_{3}$ towards isoprene is greater than its response towards acetone. This difference in selectivity from acetone to isoprene for the (100) and (001) facet texturing is likely attributed to the different surface properties of the respective planes. 

**Figure 6:**
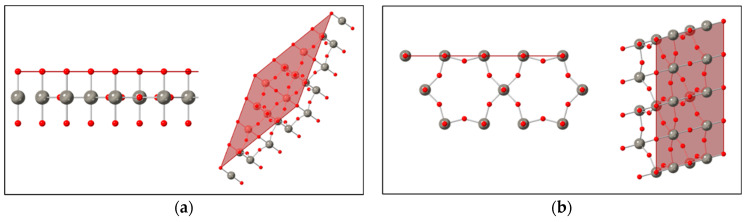
Geometrical structures of the (001) terminated surface (**a**) and the (100) terminated surface (**b**) from *h*-WO3. The crystal structure is ICSD 32001 from Gerand et al. Ref [3]. Red spheres indicate oxygen atoms, while green spheres indicate tungsten atoms. The viewing directions for the structures from left to right are [010], [111], [001], and [101].

Figure 6a shows the (001) terminated surface *h*-$WO_{3}$ structure down the [010] direction and down the [111] direction. This is compared with the (100) terminated surface in Figure 6b down the [001] and [101] viewing directions. For a defect free surface, the (001) surface has only oxygen dangling bonds, whereas the (100) surface has both oxygen and tungsten dangling bonds. Furthermore, for a super cell of identical size, the (100) terminated surface has a higher density of dangling bonds. 

**Figure 7:**
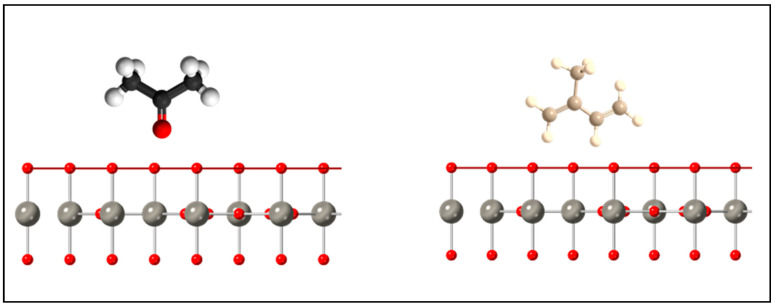
Potential molecule interaction orientation with the (001) facet of the *h*-$WO_{3}$ structure. Red horizonal line indicates the (001) plane. Red and grey spheres represent oxygen and tungsten atoms, respectively.

**Figure 8:**
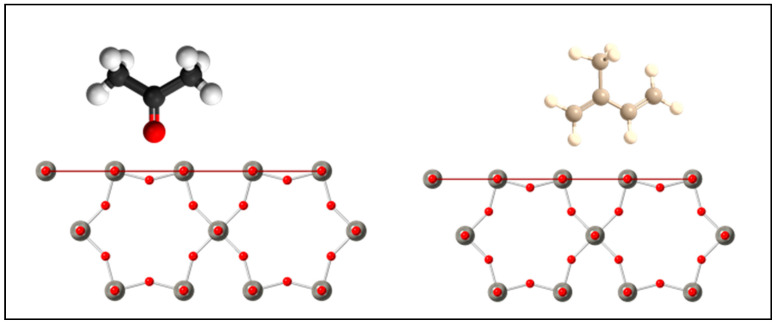
Potential molecule interaction orientation with the (100) facet of the *h*-$WO_{3}$ structure. Red horizonal line indicates the (100) plane. Red and grey spheres represent oxygen and tungsten atoms, respectively.

Looking at Figure 7 and Figure 8, the structure of the acetone molecule contains an electrical dipole moment pointing towards a lone oxygen atom. The (001) facet of the *h*-$WO_{3}$ contains mostly oxygen dangling bonds. As such, the electronegative interaction between the oxygen dangling bonds and the oxygen dipole would discourage surface interactions across the large (001) faceted surface, which accounts for most of the surface area of the material. This is supported by DFT studies, which indicate that acetone adsorption onto the (001) surface of *h*-$WO_{3}$ occurs more favorably through carbon–hydrogen bridging through the oxygen dipole on oxygen deficient surfaces where oxygen vacancies are present [24]. For the (100) facet, one can immediately see a higher density of dangling bonds as well as the presence of tungsten and oxygen dangling bonds. In particular, 

One can see the presence of a bridging oxygen dangling bond, which has been known to be a preferential interaction site for oxygen containing molecules such as acetone [25]. This increase in overall density of dangling bonds on the (100) surface likely attributes to the overall higher response of the $Na_{2}{\mathrm{WO}}_{4}\cdot 2H_{2}O$ *h*-$WO_{3}$ to acetone compared to the $WCl_{6}$ *h*-$WO_{3}$. Isoprene, while containing carbon and hydrogen atoms, lacks this oxygen containing dipole, which limits its potential adsorption configuration. As such, the material shows a response to isoprene molecule, but a significantly stronger response towards that of the acetone molecule [25,26].

In contrast, the (001) surface, which only contains oxygen dangling bond, and the isoprene molecule, which has only carbon and oxygen atoms, have a wider variety of orientational adsorption [24]. This mechanism is analogous to our previously reported gas sensing mechanism of the ferroelectric ε-$WO_{3}$ towards the acetone molecule via its ferroelectric dipole interactions with the polar acetone molecule [19]. As the temperature of the sample increases, acetone desorbs more rapidly from the surface, resulting in a decrease in response as the temperature increases [24]. This has been observed in Figure 5 and is consistent with the reports by other researchers [4,27]. The results reported herein of the (100) facet having a higher response to acetone than the (001) facet differ from the work previously reported by Jia et al., who reported a higher response for the (002) facet as opposed to the (100) facet [11]. However, it should be noted that Jia et al. synthesized hexagonal nanorods, whereas the nanomaterials studied here have platelet shapes. Furthermore, as their paper was focused mainly on ethanol and acetone, they did not report any sensing results on isoprene. It is quite possible that if tested, isoprene would have responded better for their (001) material, as ours did. 

## 5. Conclusions

Two morphologies of *h*-WO3 have been synthesized via a solvothermal and hydrothermal technique. The XRD patterns of the solvothermal material synthesized using $WCl_{6}$ were dominated by (001) facet texturing and exhibited a preferred orientation (001). The XRD pattern of the hydrothermal material synthesized using $Na_{2}{\mathrm{WO}}_{4}\cdot 2H_{2}O$ was dominated by (100) facet texturing and exhibited a higher relative intensity of the 100 diffraction peak. Both samples exhibited a platelet like morphology, and the SAED pattern of the $WCl_{6}$ shown confirms the presence of a large (001) terminated surface. The gas sensing behavior of the two materials were conducted at 250, 300, and 350 °C. At 250 °C, the $WCl_{6}$ *h*-$WO_{3}\cdot$showed better selectivity towards acetone. Upon increasing the temperature towards 300 °C, the selectivity switched from acetone to isoprene, indicating an increased desorption rate of acetone, which is consistent with the literature. At 350 °C, the $WCl_{6}$ *h*-$WO_{3}$ showed an overall lower response to both gases than that of the $Na_{2}{\mathrm{WO}}_{4}\;2H_{2}O$ *h*-$WO_{3}$. It is proposed herein that the cause for this increased selectivity and decreased response is related to the dangling bond configuration of the (001) terminated surface, which contributed to the higher response of the $WCl_{6}$ *h*-$WO_{3}$ over that of the $Na_{2}{\mathrm{WO}}_{4}\cdot 2H_{2}O$ due to the electronegativity of the surface oxygen and the acetone oxygen dipole. This is contrasted by the isoprene molecules structure, which is less polar and only contains carbon and hydrogen atoms, allowing for a lower adsorption rate. Future work in this area will revolve around improving the overall response of the $WCl_{6}$ *h*-$WO_{3}$ by optimizing the synthesis parameters and decreasing the overall particle size to increase the overall performance of the sensor. Overall, the work herein serves as an excellent step towards improving the development of *h*-$WO_{3}$ towards isoprene for the development of a flu breathalyzer. 

## Figures and Tables

**Table 1:** Texture coefficients for the 001 and 100 facets of the $Na_{2}{\mathrm{WO}}_{4}\cdot 2H_{2}O$ and $WCl_{6}$ synthesized *h*-$WO_{3}$ samples.

Sample	$TC_{001}$	$TC_{100}$
$WCl_{6}$* h*- $WO_{3}$	0.75	0.25
$Na_{2}{\mathrm{WO}}_{4}\cdot 2H_{2}O$* h*- $WO_{3}$	0.34	0.66

## Supplementary Materials

The following are available online at https://www.mdpi.com/1424-8220/21/5/1690/s1: Figure S1: (a,b) TEM micrographs of the $Na_{2}{\mathrm{WO}}_{4}\cdot 2H_{2}O$ h-$WO_{3}$ sample under different magnification. The red region in Figure S1b contains polytypic features.
