# Supplementary material for: Effect of (100) and (001) Hexagonal WO3 Faceting on Isoprene and Acetone Gas Selectivity
Source: Sensors (Basel). 2021 Mar 1;21(5):1690. doi: 10.3390/s21051690 (PMC7957717; doi:10.3390/s21051690)
Supplement: Supplementary file 1 [file sensors-21-01690-s001.pdf]

Isoprene Gas Sensor Based on (001) Faceted Hexagonal WO_3

Owen O. Abe [1], Zanlin Qiu [1], Joerg R. Jinschek [1], and Pelagia-Irene Gouma [1,2,*]

[1] Department of Materials Science and Engineering, The Ohio State University, Columbus, OH 43202, USA; abe.24@osu.edu (O.A); qiu.512@osu.edu (Z.Q); jinschek.1@osu.edu (J.J); gouma.2@osu.edu (P.G.)

[2] Department of Mechanical and Aerospace Engineering, The Ohio State University, Columbus, OH 43202, USA; gouma.2@osu.edu

* Correspondence: gouma.2@osu.edu (P.G); Tel.: +1-614-292-4391

Supplemental

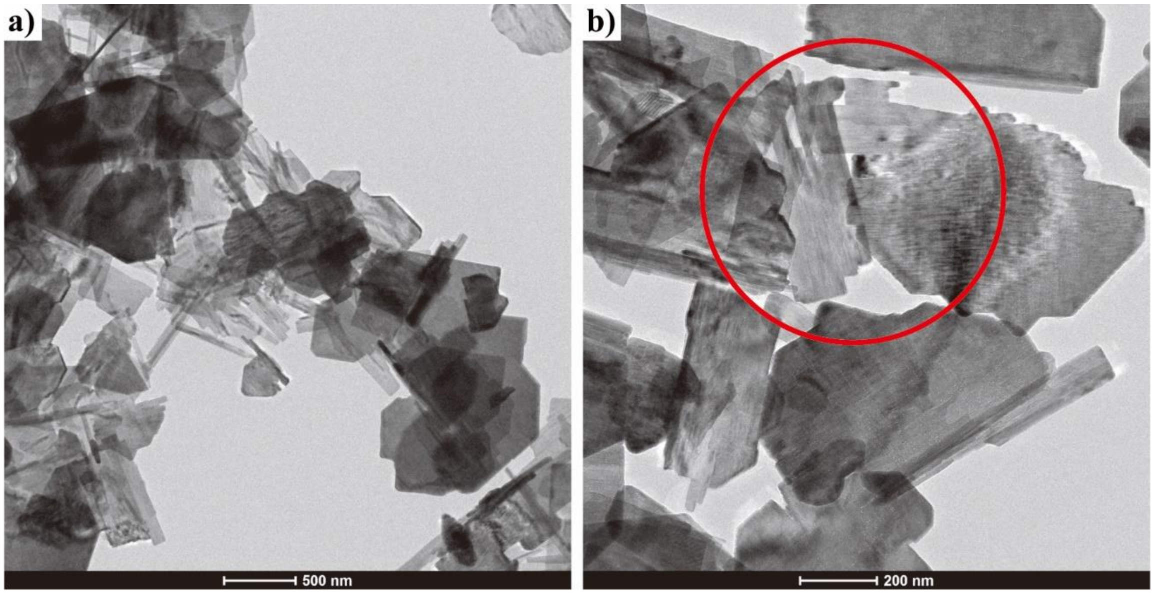

Figure S1 (a) and (b) TEM images of the $Na_2WO_4 \cdot 2H_2O$ sample under different magnification. The red region in Figure S1(b) contains polytypic features.
